# Evaluation of Different PCR-Based Assays and LAMP Method for Rapid Detection of *Phytophthora infestans* by Targeting the *Ypt1* Gene

**DOI:** 10.3389/fmicb.2017.01920

**Published:** 2017-10-05

**Authors:** Mehran Khan, Benjin Li, Yue Jiang, Qiyong Weng, Qinghe Chen

**Affiliations:** ^1^State Key Laboratory of Ecological Pest Control for Fujian and Taiwan Crops, Fujian Agriculture and Forestry University, Fuzhou, China; ^2^Fujian Key Laboratory for Monitoring and Integrated Management of Crop Pests, Institute of Plant Protection, Fujian Academy of Agricultural Sciences, Fuzhou, China

**Keywords:** LAMP, *Phytophthora infestans*, real-time PCR, sensitivity, specificity, *Ypt1*

## Abstract

Late blight, caused by the oomycete *Phytophthora infestans*, is one of the most devastating diseases affecting potato and tomato worldwide. Early diagnosis of the *P. infestans* pathogen causing late blight should be the top priority for addressing disease epidemics and management. In this study, we performed a loop-mediated isothermal amplification (LAMP) assay, conventional polymerase chain reaction (PCR), nested PCR, and real-time PCR to verify and compare the sensitivity and specificity of the reaction based on the *Ypt1* (Ras-related protein) gene of *P. infestans.* In comparison with the PCR-based assays, the LAMP technique led to higher specificity and sensitivity, using uncomplicated equipment with an equivalent time frame. All 43 *P. infestans* isolates, yielded positive detection results using LAMP assay showing no cross reaction with other *Phytophthora* spp., oomycetes or fungal pathogens. The LAMP assay yielded the lowest detectable DNA concentration (1.28 × 10^-4^ ng μL^-1^), being 10 times more sensitive than nested PCR (1.28 × 10^-3^ ng μL^-1^), 100 times more sensitive than real-time PCR (1.28 × 10^-2^ ng μL^-1^) and 10^3^ times more sensitive than the conventional PCR assay (1.28 × 10^-1^ ng μL^-1^). In the field experiment, the LAMP assay outperformed the other tests by amplifying only diseased tissues (leaf and stem), and showing no positive reaction in healthy tissues. Overall, the LAMP assay developed in this study provides a specific, sensitive, simple, and effective visual method for detection of the *P. infestans* pathogen, and is therefore suitable for application in early prediction of the disease to reduce the risk of epidemics.

## Introduction

Late blight, caused by the oomycete *Phytophthora infestans*, is one of the most devastating diseases affecting tomato and potato worldwide (Erwin and Ribeiro, 1996; Fry, 2008). During the past two decades, the disease has been increased immensely world-wide (Fry and Goodwin, 1997; Fry et al., 2013, 2015), and it is virtually ubiquitous in areas where potato crops are grown (O’Brien et al., 2009). During the growing season, the pathogen can kill foliage and stems of potato and tomato plants, and may infect potato tubers and tomato fruits, which will then rot either in the field or in storage (Lamour and Kamoun, 2009). Managing late blight epidemics have become more difficult world-wide because the pathogen has an extremely high reproductive potential and spreads at a rapidly accelerating rate. Once sporangia disperse, they can germinate within a few hours, given sufficient moisture; epidemics are rapid and devastating. Management strategies and forecasting systems in susceptible areas depend on the early diagnosis and detection of pathogens in the field.

Monitoring the extend and epidemics of *P. infestans* in the field requires quick, cost-effective and efficient detection methods. Culture-based techniques are used as standard for the detection of *P. infestans* in plant tissues; however, traditional detection methods used for identifying plant pathogens are time taking, laborious, and substantial expertise is required to distinguish species based on their morphological distinctiveness. Molecular technologies, such as polymerase chain reaction (PCR) and real-time PCR assays, have been applied successfully to detect *P. infestans* (Judelson and Tooley, 2000; Hussain et al., 2005; Lees et al., 2012). These technologies might not be existing in resource-poor regions because they require specialized laboratories, trained personnel, complicated equipment, and expensive reagents. Therefore, it is crucial to establish a simple, rapid, sensitive, and precise approach for the detection of *P. infestans* in the field.

Loop-mediated isothermal amplification (LAMP) is a promising substitute to conventional DNA-based diagnostic techniques due to its high sensitivity and specificity, and capability to yield quick results (Notomi et al., 2000; Francois et al., 2011). This technique uses DNA amplification with *Bst* polymerase, which reveals strand displacement activity by amplifying target DNA with four to six primers under isothermal conditions. The LAMP assay can amplify large quantities of DNA under isothermal conditions, having temperature range of 60–65°C for not more than 60 min (Notomi et al., 2000). LAMP products can be observed by agarose gel electrophoresis stained with ethidium bromide, and visualized via the naked eye by adding intercalating dyes like SYBR Green or calcein (Niu et al., 2012).

Selection of a suitable gene for primer and probe design is crucial for nucleic acid sequence-based diagnostic methods. The internal transcribed spacer (ITS) areas of the nuclear-encoded ribosomal RNA genes (rDNA) are broadly used to recognize and detect *Phytophthora* species (Cooke et al., 2000; Silvar et al., 2005; Pavón et al., 2008), and *P. infestans*-specific primers based on the ITS for LAMP detection have also been used in diseased plant tissues, soil, and water to rapidly detect the pathogen (Hansen et al., 2016). However, they are not adequately flexible to separate closely related taxa frequently (Kroon et al., 2004; Schena and Cooke, 2006; Chen et al., 2013; Li et al., 2013). For this reason, recently reported studies have observed the Ras-related protein gene *Ypt1*, β-tubulin, elicitin, and the spacer region amongst the mitochondrially encoded cox1 and cox2 genes in some *Phytophthora* spp. (Schena and Cooke, 2006; Schena et al., 2008; Meng and Wang, 2010; Martin et al., 2012). The elicitin gene *parA1* and putative storage protein gene (*Lpv*) have been useful targets for the precise identification of *P. cinnamomi* and *P. nicotianae*, respectively (Kong et al., 2003a,b). However, neither gene contains introns, nor is likely to be suitably variable to differentiate among a wide range of species (Schena and Cooke, 2006). The *Ypt1* (Ras-related protein gene) contains sufficient variation, having coding (exons) and non-coding (UTRs and introns) loci suitable for the developing molecular markers for nearly all *Phytophthora* species, without intra-specific variability (Haubruck et al., 1987; Schena and Cooke, 2006; Schena et al., 2008; Chen et al., 2013; Li et al., 2013).

The objective of this study was to develop a LAMP technique for the detection of *P. infestans* by targeting the *Ypt1* gene in plant tissues. We conducted different types of PCR-based assays for specificity and sensitivity to compare with the results of the LAMP assay to evaluate the best detection method. The LAMP assay provided a useful tool for the diagnosis of *P. infestans* infection.

## Materials and Methods

### Source of Isolates

We examined 43 isolates of *P. infestans* randomly collected from different geographic areas in China, 29 isolates from 15 different *Phytophthora* spp., along with 15 isolates from other oomycetes and fungi. The origin, host, and number of isolates examined in current study are listed in **Table 1**.

**Table 1:** Isolates used in this study.

				Result^b^		
Species^a^	Host	Number of isolates	Source	Yph1F/Yph2R	Nested PCR	LAMP
*Phytophthora infestans*	*Solanum tuberosum*	5	Fujian, China	+	+	+
*Phytophthora infestans*	*Solanum tuberosum*	5	Inner Mongolia, China	+	+	+
*Phytophthora infestans*	*Solanum tuberosum*	6	Hubei, China	+	+	+
*Phytophthora infestans*	*Solanum tuberosum*	3	Ningxia, China	+	+	+
*Phytophthora infestans*	*Solanum tuberosum*	3	Yunnan, China	+	+	+
*Phytophthora infestans*	*Solanum tuberosum*	5	Heilongjiang, China	+	+	+
*Phytophthora infestans*	*Lycopersicon esculentum*	5	Guangxi, China	+	+	+
*Phytophthora infestans*	*Lycopersicon esculentum*	4	Fujian, China	+	+	+
*Phytophthora infestans*	*Lycopersicon esculentum*	3	Jiangsu, China	+	+	+
*Phytophthora infestans*	*Lycopersicon esculentum*	4	Jilin, China	+	+	+
*Phytophthora cactorum*	*Malus pumila*	2	Fujian, China	+	_	_
*Phytophthora nicotianae*	*Nicotiana tabacum*	3	Fujian, China	+	_	_
*Phytophthora capsici*	*Capsicum annuum*	4	Fujian, China	+	_	_
*Phytophthora melonis*	*Cucumis meloa*	2	Fujian, China	+	_	_
*Phytophthora sojae*	*Glycine max*	5	Fujian, China	+	_	_
*Phytophthora vignae*	*Vigna unguiculata*	2	Fujian, China	+	_	_
*Phytophthora parasitica*	*Nicotiana tabacum*	3	Fujian, China	+	_	_
*Phytophthora drechsleri*	*Beta vulgaris*	1	Fujian, China	+	_	_
*Phytophthora boehmeriae*	*Gossypium*	1	Jiangsu, China	+	_	_
*Phytophthora citrophthora*	*Citrus reticulata*	1	Fujian, China	+	_	_
*Phytophthora citricola*	*Citrus reticulata*	1	Fujian, China	+	_	_
*Phytophthora botryosa*	*Colocasia esculenta*	1	Fujian, China	+	_	_
*Phytophthora cinnamomi*	*Cedrus deodara*	1	Fujian, China	+	_	_
*Phytophthora cryptogea*	*Gerbera jamesonii*	1	Fujian, China	+	_	_
*Phytophthora fragariae*	*Arbutus menziesii*	1	Fujian, China	+	_	
*Peronophthora litchi*	*Litchi chinensis*	1	Fujian, China	+	_	_
*Pythium aphanidermatum*	*Cucumis sativus*	1	Fujian, China	+	_	_
*Alternaria alternata*	*Solanum tuberosum*	1	Fujian, China	_	_	_
*Colletotrichum gloeosporioides*	*Solanum tuberosum*	1	Fujian, China	_	_	_
*Rhizoctonia solani*	*Solanum tuberosum*	1	Fujian, China	_	_	_
*Ralstonia solanacearum*	*Solanum tuberosum*	1	Fujian, China	_	_	_
*Erwinia carotovora*	*Solanum tuberosum*	1	Fujian, China	_	_	_
*Verticillium dahliae*	*Solanum melongena*	1	Fujian, China	_	_	_
*Magnaporthe oryzae*	*Oryza sativa*	1	Fujian, China	_	_	_
*Fusarium oxysporum*	*Gossypium hirsutum*	1	Fujian, China	_	_	_
*Fusarium moniliforme*	*Gossypium hirsutum*	1	Fujian, China	_	_	_
*Fusarium graminearum*	*Triticum aestivum*	1	Fujian, China	_	_	_
*Botryosphaeria rhodina*	*Psidium guajava*	1	Fujian, China	_	_	_
*Helminthosporium turcicum*	*Zea mays*	1	Fujian, China	_	_	_
*Sclerotinia sclerotiorum*	*Glycine max*	1	Fujian, China	_	_	_

^a^*All isolates were maintained in the collection of Fujian Academy of Agricultural Sciences.*

^b^*Presence (+) or absence (-) are based on the presence of a PCR or LAMP result of the expected size.*

### Culture Conditions and DNA Extraction

The isolates and fungi were carefully amended on rye agar (Erwin and Ribeiro, 1996) or potato dextrose agar. Pathogen mycelia were grown in potato dextrose broth at 18–20°C (temperature varied depending on the pathogen) for 4–5 days, and mycelia were harvested and freeze dried. Total DNA extraction was done via DNA extraction kit (BioTeke Corp., Beijing, China). Extracted DNA concentration was checked using a NanoDrop 2000 (Thermo Fisher Scientific, Waltham, MA, United States) and aliquots were diluted to 100 ng/μL using RNA–free, sterilized with double-distilled water, followed by storing at -20°C.

### Total DNA Extraction from Infected Plant Samples

Total DNA was extracted from *P. infestans*-infected potato tissues (leaf and stem) with the Cetyl-Trimethylammonium Bromide (CTAB) procedure, as described earlier (Zhang et al., 2006). DNA samples from infected potato tissues were extracted rapidly, as described previously (Tooley et al., 1997) but with minor modifications. A 10-mg sample from a diseased potato plant (stem or leaf) and healthy tissues were excised and added to freshly prepared 0.5 M NaOH with a volume of 10 μl in each 1.5 mL EP (Eppendorf tube). The samples were crushed with a glass pestle and then subjected to centrifugation for approximately 5 min at 12,000 × *g*. After centrifugation, 5 μL of the liquid phase was diluted directly with 195 μL of 100-mM Tris (pH 8.0 adjusted). The extracted DNA samples were used directly for the LAMP reaction, or were frozen at -20°C for future usage. The products were tested further by electrophoresis in 2% agarose gel stained with ethidium bromide.

### Primer Design for *P. infestans*

**FIGURE 1:**
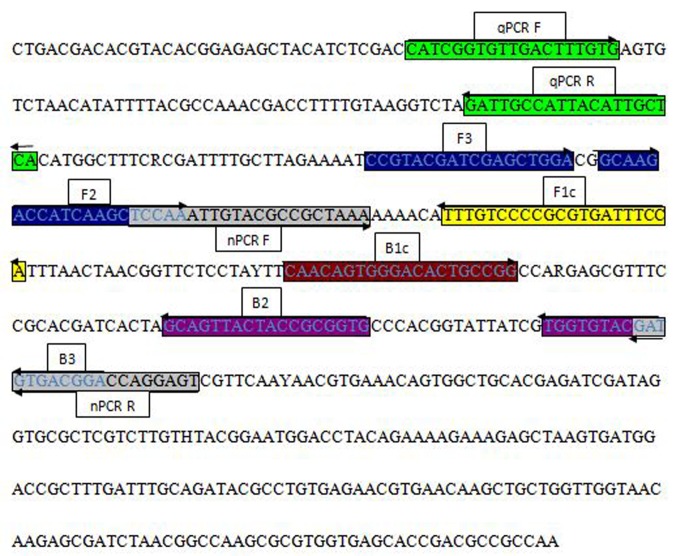
Sequence and location of the *P. infestans Ypt1* gene used to design polymerase chain reaction (PCR) and loop-mediated isothermal amplification (LAMP) primers. Primer locations for PCR (qPCRF, qPCRR; nPCRF, nPCRR) and LAMP (F3, B3, FIP [F1c-F2], BIP [B1c-B2]) techniques. FIP is a hybrid primer consisting of the F1c and F2 sequences; BIP is a hybrid primer consisting of the B1c and B2 sequences. Primer sequence sites are underlined. Arrows indicate the extension direction.

In this study, *Ypt1* (Ras-related protein) was used as a target gene to detect *P. infestans* isolates collected from various regions of China. We collected sequence information for *P. infestans* in the *Ypt1* gene, which had been analyzed in a previous study and documented in GenBank (accession number JN678988); we then performed a BLAST search of the National Center for Biotechnology Information (NCBI) database using sibling species to identify the *Ypt1* conserved region. PCR universal primers, comprising forward and backward primers, were selected from a previous study of *Phytophthora* spp. based on sibling target genes (Chen et al., 2013). The *P. infestans* fragments that were isolated from pure cultures of the same species were cloned and sequenced. Based on the *P. infestans Ypt1* target sequence, four LAMP primers were designed, consisting of two outer primers (F3 and B3) and two inner primers (FIP and BIP) using PrimerExplorer V4 software^1^ (Eiken Chemical Co., Tokyo, Japan) as shown in **Figure 1**. Primers for nested PCR were constructed via Primer Premier 5 software, and real-time PCR primers were constructed using Beacon Designer software with the *Ypt1*-based sequence. Information on the primers used in the current study is presented in **Table 2** and **Figure 1**.

**Table 2:** Primers used in this study.

Primer	Type primer	Primer sequences (5′ to 3′)
Universal primers	Yph1F	CGACCATKGGTGTGGACTTT
	Yph2R	ACGTTCTCMCAGGCGTATCT
Nested PCR	nPCR F	TCCAAATTGTACGCCGCTAAA
	nPCR R	ACTCCTGGTCCGTCACATC
Real-time PCR	qPCR F	CATCGGTGTTGACTTTGTG
	qPCR R	TGAGCAATGTAATGGCAATC
LAMP	F3	CCGTACGATCGAGCTGGA
	B3	CACCGCGGTAGTAACTGC
	FIP	TGGAAATCACGCGGGGACAAA- GCAAGACCATCAAGCTCCAA
	BIP	CAACAGTGGGACACTGCCGG- CACCGCGGTAGTAACTGC

### PCR Detection of *P. infestans*

The reaction mixtures (final volume: 25 μL) consisted of 12.5 μL of 2× TaqPCR MasterMix (TIANGEN, Beijing, China), 10 pmol of each primer, 1 μl of 100-ng template DNA, and sterilized double distilled water to create a final volume of 25 μl. All reactions were completed in a PTC200 Thermo Cycler (MJ Research, Watertown, MA, United States) and preset for an initial denaturation step at 95°C for 3 min, followed by 30 cycles of denaturation with a temperature of 95°C for 1 min, annealing for 1 min at 54°C, extension for 1 min at 72°C, with a final extension temperature of 72°C for 5 min to evaluate the universal primers (Yph1F/Yph2R). Nested PCR consisted of two amplification rounds, thereby using *Phytophthora* spp. universal primers (Yph1F/Yph2R) as outer primers in the first round and *P. infestans*-specific primers nPCRF/nPCRR were used in the second amplification round (**Table 2**); by using 1 μL of amplified product from the first round as a template DNA by amplifying a product of 203-bp. The second round PCR protocol started with a preliminary denaturation step at 95°C for 3 min, followed by 32 cycles of denaturation for 1 min at 95°C, annealing for 1 min at 66°C, extension for 1 min at 72°C, and a final extension temperature of 72°C for 5 min. The sensitivity of primer pairs from different PCRs was tested using 10-fold serial dilution of the template DNA, ranging from 100 ng to 10 fg of isolated, purified DNA as a template. Negative controls devoid of template DNA were used in each experiment to check for contaminated reagents. All reagents used in this experiment for PCR amplification were purchased from TaKaRa Bio Inc. (Dalian, China). All products were then subjected to electrophoresis on 2% gel stained with ethidium bromide. All experiments were repeated at least three times.

### Real-time PCR Specificity and Sensitivity

All quantitative real-time PCR assay reactions were performed using a CFX96 real-time detection system (Bio-Rad, Hercules, CA, United States) by adding Maxima^TM^ SYBR Green I qPCR Master Mix (Purchased from TaKaRa, Dalian, China) in 0.5-ml thin-walled, optical grade PCR tubes for a final reaction volume of 25 μl. The reaction mixture consisted of 12.5 μl of Maxima^TM^ SYBR Green I qPCR Master Mix, 1 μl (10 μM) of each primer (qPCR F and qPCR R), along with 1 μl of DNA template, with sterilized double distilled water added to create the final volume. The amplification conditions for the reaction were 95°C for 30 s, with 40 cycles of 95°C for 5 s, followed by 60°C for 30 s, 72°C for 20 s, and a fluorescence read at 72°C at the end of each cycle, with a final melting curve at 65–95°C with an addition of 0.1°C s^-1^. DNA was serially diluted using Easy Dilution (TaKaRa) to calculate the amplification efficiency of sensitivity with an estimated initial concentration of 1.28 × 10^2^ ng μL^-1^, diluted in a 1:10 series from 1.28 × 10^2^ ng μL^-1^ to 1.28 × 10^-4^ ng μL^-1^. The results of the assay were analyzed by plotting the log of the template DNA concentration against cycle threshold (*C*_t_) values, using the formula *E* = [10 (-1/slope) - 1] × 100 to calculate the PCR efficiency, where *E* is the amplification efficiency and the slope is derived from the plot of log of the template DNA concentration against *C*_t_. Values of *C*t, standard curves, and corresponding correlation coefficients (*R*^2^) were generated automatically by Sequence Detection System v.1.2 software, by interpolating *C*_t_ values against the logarithm of the initial DNA concentrations.

### Optimization of the LAMP Assay

To identify the optimal reaction for visual LAMP amplification, a range of concentrations of FIP/BIP (0.8–2.4 mM), F3/B3 (0.1–0.5 mM), MgSO_4_ (2–10 mM), dNTPs (0.2–2 mM), and Betaine (0.8–1.6 M) (Sigma–Aldrich, St. Louis, MO, United States), and different incubation times (30–70 min) and temperatures (57–67°C) were used. The LAMP reaction system used in this study consisted of 12.5 μl 2 × LAMP Buffer [10xThermopol Buffer, dNTPs (25 mM), Betaine (5 M), MgSO_4_ (50 mM), and Tween20 (2.5%)], 4 μl of FIP/BIP and F3/B3 primers, 1 μl of 50 μM Calcein-500 μM MnCl_2_, 1 μL of 8000 U/ml *Bst* DNA Polymerase (Purchased from *BioLab^®^ Inc.*, New England), 1 μL of template DNA (100 ng/μL) and double distilled water to make the final volume of 25 μL. The LAMP products were visually inspected by naked-eye or by running gel electrophoresis to conclude the optimal reaction conditions. All reactions were performed in 0.2-mL sterilized microtubes in a temperature-controlled water bath. A positive reaction yielded a green color, and a negative reaction yielded a brown color, through the quenching effect of 50 μM Calcein-500 μM MnCl_2_. Nuclease-free water was used in negative reaction tubes instead of DNA.

### LAMP Assay Specificity

To determine the specificity of LAMP for the target *Ypt1* gene, 43 isolates of *P. infestans* from different geographic areas, 29 isolates from 15 different *Phytophthora* spp., and 15 other oomycetes and fungi (**Table 1**) were subjected to LAMP assays. The amplified LAMP products were detected as described above. All the experiments in the present study were repeated at least three times.

### LAMP Assay Sensitivity vs. Nested PCR Sensitivity

To determine the sensitivity of LAMP, 10-fold dilutions ranging from 1.28 × 10^2^ to 1.28 × 10^-4^ ng μL^-1^ (1.28 × 10^2^ ng μL^-1^; 1.28 × 10^1^ ng μL^-1^; 1.28 ng μL^-1^; 1.28 × 10^-1^ ng μL^-1^; 1.28 × 10^-2^ ng μL^-1^; 1.28 × 10^-3^ ng μL^-1^; 1.28 × 10^-4^ ng μL^-1^) of purified *P. infestans* DNA were prepared. Based on the *Ypt1* gene sequence, we designed a specific nested PCR primer pair, nPCR-F/nPCR-R, and the sensitivity of the primers was evaluated to compare the LAMP assays. Nested PCR was performed as described above using a PTC-200 PCR apparatus (MJ Research). The LAMP amplification products were detected as described above. Nested PCR amplification products were separated by electrophoresis on 2.0% agarose gels stained with ethidium bromide, and visualized under ultraviolet (UV) light. All experiments carried out were repeated at least three times.

### Early Detection of *P. infestans* by the LAMP Assay

An infection time course experiment was performed to evaluate the earliest time point when *P. infestans* was detected by LAMP. Potato plants were grown in a greenhouse in plastic pots filled with a mixture of equal proportions of loamy soil, sand and composted farmyard manure. The plants were maintained at a mean daily temperature of 22°C under a 12-h day photoperiod. A suspension of *P. infestans* sporangia were made consisting of 10 sporangia/μL (Hussain et al., 2005). The leaves of the 6-week-old each potato plant was spray inoculated with 50 μL of the suspension (10 sporangia/μL) allowing them to stay at room temperature for different time span (1, 2, 4, 8, 16, and 32 h) along with negative control using double distilled water inoculation. After the given time span, the leaves was detached and washed thoroughly with tap water and rinsed three times with sterile distilled water. Total DNA was extracted rapidly from plant tissues as described above (Tooley et al., 1997). The DNA extracted was then subjected to LAMP assays. The amplified LAMP products were detected as described above. All the experiments were repeated at least three times.

### Evaluation of *P. infestans* DNA from Diseased Plant Tissues by LAMP Assay

To evaluate the application of LAMP for *P. infestans* detection in field sample, healthy-looking (symptomless) but pathogen-infected potato were collected from three different locations for LAMP detection. Total DNA was extracted from diseased plant tissues (potato leaf and stem) following the CTAB procedure, with fast DNA extraction via the NaOH procedure described above. Healthy plant tissues were used as controls, and LAMP reactions were performed with a constant temperature of 65°C for 60 min in water bath. Amplified products were observed using 2% agarose gel electrophoresis. *P. infestans* infection was confirmed using the conventional culture method for the identification of plant pathogens based on morphological characteristics (Fry, 2008). All the experiments conducted were repeated at least three times.

## Results

### Optimization of LAMP Assay

We performed the LAMP assay using *P. infestans* DNA as a template to determine the optimum primer concentrations, time, temperature, and amount of dNTPs, betaine, and MgSO_4_. The results revealed an optimal temperature of 65°C, and an optimal time of 60 min. In our experiment, 1.5 μM FIP/BIP primers, 0.7 mM dNTPs, 0.8M betaine, and 4 mM MgSO_4_ represented the best conditions. As expected, a ladder-like pattern on 2% agarose gel was observed for all isolates showing a positive reaction, but no such pattern was seen for negative reactions (**Supplementary Figure S1**). The characteristics of the products amplified were confirmed by sequencing, as the sequences obtained perfectly matched the expected DNA sequences of *P. infestans* (data not shown). Visual inspection of LAMP products revealed a green color for all positive reactions and a brown color for negative reactions (**Supplementary Figure S1**). Green color appeared through the addition of Calcein-MnCl_2_ in the LAMP assay due to its quenching effect. All reactions in this assay were performed at least three times.

### Specificity of *P. infestans* Using LAMP and PCR Assays

**FIGURE 2:**
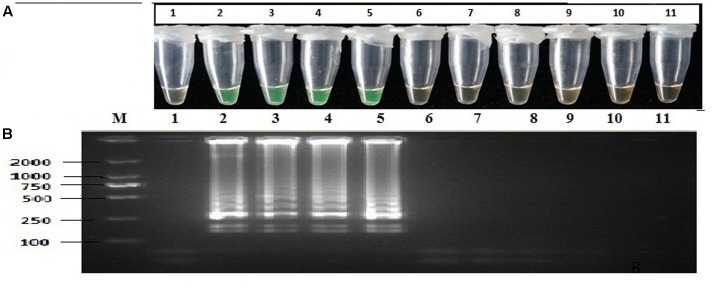
Specificity of LAMP detection of *P. infestans*. Assessment was based on **(A)** calcein visualization of color change, **(B)** agarose gel electrophoresis analysis of the LAMP products. Lane 1: negative control; Lanes 2–5: *P. infestans* from different geographic areas; Lane 6: *P. cactorum*; Lane 7: *P. nicotianae*; Lane 8: *P. capsici*; Lane 9: *P. melonis*; Lane 10: *P. sojae*; Lane 11: *P. boehmeriae*; Lane M: DL2000 DNA markers. The same results were obtained in three repeat assessments.

The specificity of the reaction was confirmed by direct visual observation through the addition of Calcein-MnCl_2_ during the reaction, and the same products were confirmed for LAMP in electrophoresis in 2% agarose gel stained with ethidium bromide. The results showed that LAMP *Ypt1* primer amplification yielded positive reactions when tested with 43 different isolates of *P. infestans* collected from different geographic areas in China; the same LAMP *Ypt1* primers could not yield positive reactions when tested with 29 isolates from different *Phytophthora* spp., including two other closely related Clade 1 species of *P. infestans*, and 15 other oomycetes and fungi; thus, the high specificity of the primers was confirmed (**Table 1** and **Figure 2**). Tubes showing a positive reaction exhibited a ladder-like structure, unlike tubes showing a negative reaction when subjected to gel electrophoresis (**Figure 2B**). All reactions were performed at least three times. Nested PCR yielded the same specificity as the LAMP assay. As expected, a positive reaction was observed for all *P. infestans* isolates; however, no positive reaction was observed for other species, oomycetes, or fungi (**Supplementary Figure S2**). The specificity of the LAMP reaction was in line with that of PCR, which was further confirmed by 2% agarose electrophoresis on gel stained with ethidium bromide. The results revealed that LAMP and PCR assays were highly specific for *P. infestans*, based on the *Ypt1* gene.

### Sensitivity of the LAMP Assay and Nested PCR

**FIGURE 3:**
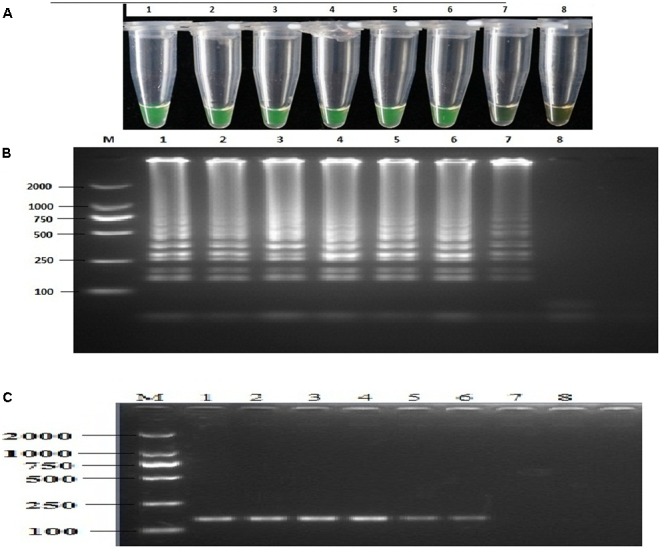
Sensitivity of the LAMP and nested PCR assays. LAMP and nested PCR assays using 10-fold serial dilutions of purified target DNA from *P. infestans* genomic DNA. **(A)** Detection of LAMP products by calcein. **(B)** Agarose gel electrophoresis analysis of the LAMP products. **(C)** Agarose gel electrophoresis analysis of the nested PCR products. Concentrations of template DNA were as follows: Lane 1, 1.28 × 10^2^ ng μL^-1^; Lane 2, 1.28 × 10^1^ ng μL^-1^; Lane 3, 1.28 ng μL^-1^; Lane 4, 1.28 × 10^-1^ ng μL^-1^; Lane 5, 1.28 × 10^-2^ ng μL^-1^; Lane 6, 1.28 × 10^-3^ ng μL^-1^; Lane 7, 1.28 × 10^-4^ ng μL^-1^; Lane 8, negative control; Lane, 2000-bp DNA marker. The same results were obtained in three repeat assessments.

Sensitivity was assessed using 10-fold serial dilution of genomic DNA by LAMP, nested PCR, and conventional PCR. The sensitivity of the LAMP assay was 1.28 × 10^-4^ ng μL^-1^ of genomic DNA per reaction. The resulting products were further analyzed through 2% agarose gel electrophoresis and visual inspection by the naked eye (**Figure 3**). Sensitivity was further assessed by nested PCR, and the primer pairs detected 1.28 × 10^-3^ ng μL^-1^ of purified DNA per 25 μL of reaction volume (**Figure 3**). Comparatively, the least detection limit for the PCR assay was 1.28 × 10^-1^ ng of genomic DNA, revealing that LAMP and the nested PCR assay were far more sensitive than the conventional PCR assay. All assays in this experiment were repeated at least three times (**Figure 3** and **Table 3**).

**Table 3:** Comparison of PCR and LAMP sensitivity in this study.

	1.28 × 10^2^	1.28 × 10^1^	1.28	1.28 × 10^-1^	1.28 × 10^-2^	1.28 × 10^-3^	1.28 × 10^-4^	1.28 × 10^-5^
	ng μL^-1^	ng μL^-1^	ng μL^-1^	ng μL^-1^	ng μL^-1^	ng μL^-1^	ng μL^-1^	ng μL^-1^
PCR	+	+	+	+	-	-	-	-
Real-time PCR	+	+	+	+	+	-	-	-
Nested PCR	+	+	+	+	+	+	-	-
LAMP	+	+	+	+	+	+	+	-

### Detection by Real-time PCR

**FIGURE 4:**
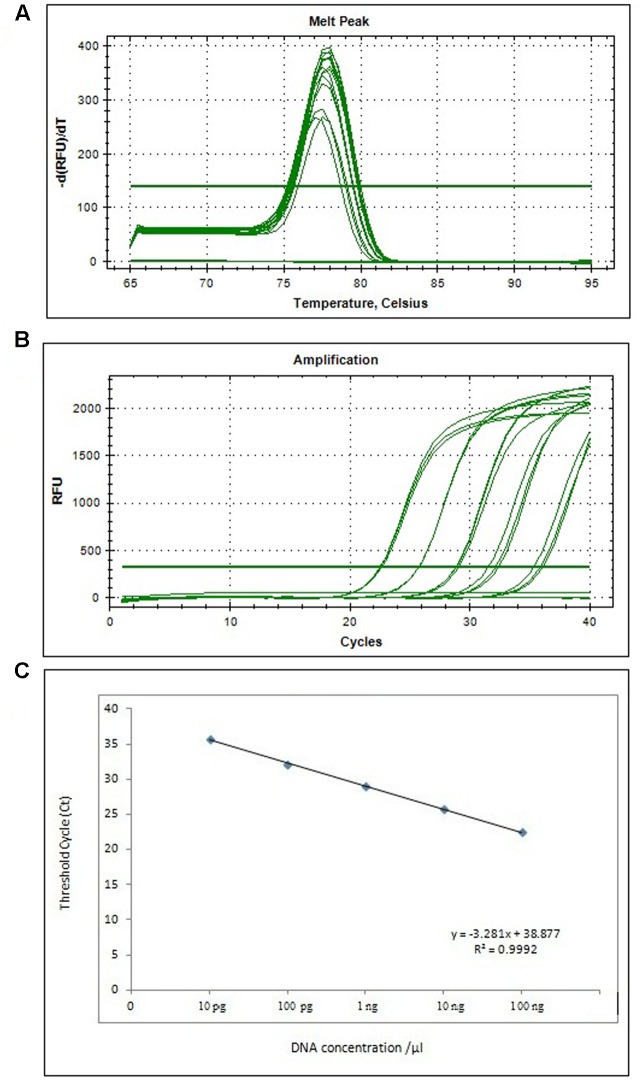
The melting curve **(A)**, amplification plot **(B)**, and standard curve **(C)** of real-time PCR for detection of *P. infestans* based on the *Ypt1* gene. **(A)** Representative melting curves using SYBR Green I for detection of *P. infestans*. **(B)** A typical amplification plot for 10-fold serial dilution containing 100 ng to 10 pg of DNA. **(C)** Standard curve derived from absolute quantification of genomic DNA yielded from 10-fold serially diluted DNA from a pure culture of *P. infestans.*

Real-time PCR reactions were performed using 10-fold dilution of target genomic DNA based on the *Ypt1* target gene, yielding to *P. infestans C*_t_ values (22.51 ± 0.08–35.75 ± 0.39) and negative control de-voiding of template DNA, thereby quantifying the specificity of the reaction. The 10-fold dilution sensitivity of template DNA determined by quantitative real-time PCR ranged from 1.28 × 10^2^ to 1.28 × 10^-2^ ng μL^-1^, amplifying the 89-bp product (**Figure 4** and **Supplementary Figure S3**); the *C*_t_ value and target DNA were linearly correlated with an extremely high coefficient of determination (*y* = –3.281*x* + 38.877, *R*^2^ = 0.9992).

### Early Detection of *P. infestans* by the LAMP Assay

To evaluate the earliest time point of detection of *P. infestans*, an time course experiment was performed after artificial infection of potato leaves with low quantities of inoculum. LAMP assays after 1, 2, 4, 8, 16, and 32 h showed that infected leaves at all different time points gave positive results, while the uninfected control showed no amplification (**Supplementary Figure S4A**). The amplified products were subjected to gel electrophoresis to further confirm the reaction shown in **Supplementary Figure S4B**. All reactions were repeated at least three times with consistent results.

### LAMP Detection of Infected Field Samples

**FIGURE 5:**
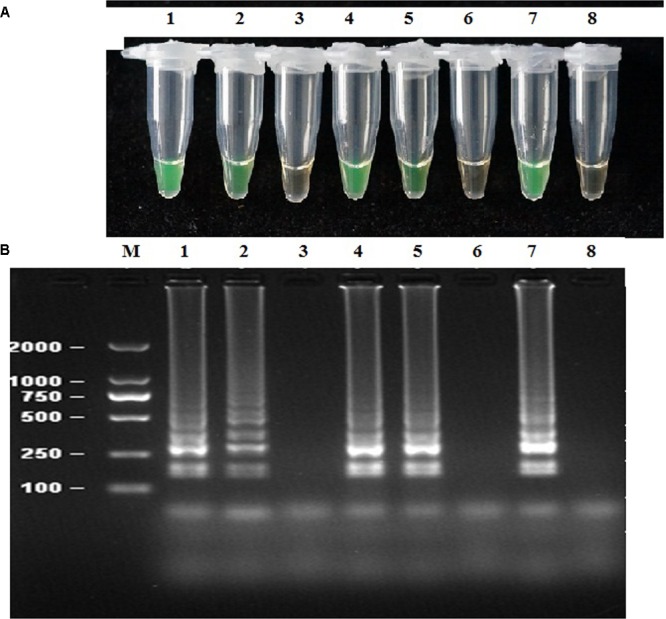
LAMP detection of *P. infestans* from infected potato plant tissues. **(A,B)** LAMP assay using two different DNA extraction methods (rapid DNA extraction and CTAB DNA extraction) from potato plant tissues (leaf and stem). Lane 1, DNA extracted by rapid extraction from infected potato leaf; Lane 2, DNA extracted from infected stem by rapid DNA extraction; Lane 3, healthy plant tissue DNA extraction by rapid DNA extraction; Lane 4, DNA extracted from infected leaf by the CTAB method; Lane 5, DNA extracted from infected stem by the CTAB method; Lane 6, DNA extracted from healthy plant tissues by the CTAB method; Lane 7, positive control; Lane 8, negative control; Lane, 2000-bp DNA marker. The same results were obtained in three repeat assessments.

To confirm the application of the LAMP assay in the field, pathogen-infected (but healthy-looking and symptomless) tissues collected from infected fields and healthy plants control were subjected to LAMP assays using two different DNA extraction methods. Both extraction methods showed positive reaction results, indicating pathogen-infected tissues and positive controls, but no reaction for healthy plants or negative controls. The positive reaction exhibited a ladder-like pattern when subjected to 2% electrophoresis on gel stained with ethidium bromide, whereas healthy plants and negative controls showed no bands, thereby confirming the specificity of the *Ypt1* primer pairs. The results of the LAMP assay and gel electrophoresis are shown in **Figure 5**. The isolation of *P. infestans* from these samples was performed using conventional isolation methods. The positive-samples (healthy-looking but pathogen-infected) were confirmed by the traditional isolation method. This was not the case for the samples in which *P. infestans* was not detected by the conventional culture method. Samples obtained from the healthy control were negative for *P. infestans*. These findings indicate the high detection competency of the LAMP assay.

## Discussion

Traditionally *P. infestans* identification, based on morphological microscopic observation and PCR-based molecular techniques, are time-consuming and requires specialized techniques and/or knowledge. The current report unveils a novel method to detect *P. infestans* using the LAMP method. LAMP reactions were completed within 60 min by incubation at 65°C, results from the study were visually observed using calcein staining, following by gel electrophoresis to confirm the amplicons. In the current study, LAMP technique showed higher sensitivity than conventional PCR, nested PCR, and real-time PCR, as it could detect 1.28 × 10^-4^ ng μL^-1^ pure genomic DNA. Furthermore, the results from the current studies indicated that the LAMP assay could be used successfully to diagnosis crude DNA extracted from plant tissue (stem and leaf) samples. Therefore, comparatively to traditional methods, the LAMP assay has the beauty of time efficiency, low cost, and ease of operation, significantly increasing the effectiveness of *P. infestans* identification and management.

From previous studies, it has been concluded that LAMP is much more sensitive than PCR; furthermore, nested PCR showed 1,000 times more sensitivity than conventional PCR (Grote et al., 2002). In our study, we assessed the sensitivity of the four methods (conventional PCR, nested PCR, real-time PCR, and the LAMP assay). Among these techniques, LAMP was highly preferred, due to its high sensitivity, specificity, rapid completion, and isothermal nature.

Traditionally, the ITS region has been used to design general primers for detection, due to its high polymorphism. However, in more recent studies, several alternative nuclear loci that have proven useful for molecular identification purposes have been sequenced for phylogenetic resolution within *Phytophthora* spp., such as the Ras-related *Ypt1* gene, 60S ribosomal protein L10, enolase, β-tubulin, large subunit rRNA, HS protein 90, Tig A gene fusion, and translation elongation factor 1α, as well as mitochondrial loci such as cox1, cox2, nad1, nad9, rps10, and secY (Meng and Wang, 2010; Martin et al., 2012; Chen et al., 2013). The Ras-related *Ypt1* gene has also been used to rapidly detect *P. nicotianae*, showing high sensitivity and specificity (Meng and Wang, 2010). The *Ypt1* region has been selected for PCR assays in previous studies because of its specificity and sensitivity (Haubruck et al., 1987; Meng and Wang, 2010). In more recent studies, the *Ypt1* LoC array has enabled important *Phytophthora* spp.-specific detection, where it did not show false positive results, unlike the ITS region, which displayed a cross reaction with non-targeted species (König et al., 2015).

To the best of our knowledge, this study is novel to select the *Ypt1* region to identify *P. infestans* using different PCR assays (conventional PCR, nested PCR, and real-time PCR), for comparison with the LAMP technique to determine the most effective method for rapid detection of pathogens. Four primers were constructed (F3/B3, FIP/BIP) based on the *Ypt1* gene sequence that was able to target six distinct regions of *P. infestans* target genomic DNA. The *Ypt1* gene showed high sensitivity and specificity in the LAMP assay (1.28 × 10^-4^ ng μL^-1^, i.e., 128 fg) in this study, unlike ITS II (2 pg), which was used to detect *P. infestans* in a previous study (Hansen et al., 2016) and yielded similar results to our nested PCR assay results. Furthermore, the *Ypt1* gene is more sensitive and specific than ITS II, which has the drawback of cross reactivity with other species, such as *P. nicotianae* (Kroon et al., 2004; Blair et al., 2008, 2012; Hansen et al., 2016). The LAMP assay conducted in this study was highly sensitive and consumed less operational time compared with conventional PCR, nested PCR and real-time PCR, amplifying the target DNA in a period of 60 min under isothermal conditions at 65°C. In this study, we found that LAMP was 10 times more sensitive than nested PCR, 10^2^ times more sensitive than real-time PCR, and 1,000 times more sensitive than conventional PCR (**Table 3**).

In this study, the earliest time point of detection of the *P. infestans* pathogen was determined by time course experiments after artificial infection. The results indicated that infected leaves at all time points, the earliest 1h after infection, showed positive results, while uninfected controls showed no amplification (**Supplementary Figure S4**). The amplification of healthy-looking but pathogen-infected plant tissues collected from natural fields also confirmed LAMP specificity by showing no amplification of healthy plant tissues using LAMP primers, and also demonstrated the ease of application of the LAMP assay in the field. A low level of pathogen latently infected potato could potentially result in late blight epidemic under favorable environmental conditions (Erwin and Ribeiro, 1996). Although fungicides and cultural practices play an important role in late blight management, they require diagnosis of the pathogen during early stages (that is healthy-looking but pathogen-infected) of disease development in potato crop production. Thus, early diagnosis, sensitive, and rapid detection of *P. infestans* is very important to control late blight in the field.

Late blight represents a major threat to potato and tomato crops worldwide, thereby emphasizing the need for a rapid and accurate method to detect and manage this devastating disease during the early stages of infection (Fry et al., 2015). Unlike PCR, LAMP results in no lost time due to temperature changes during enzymatic reactions or inhibition in later stages (Osawa et al., 2007). Late blight caused by *P. infestans* leads to significant potato and tomato crop loss, with devastating consequences for the global quality and yield of these crops. Thus, accurate and early detection of infestation by the LAMP technique allows for time-saving and sensitive detection, which has the potential to mitigate these significant losses.

## Author Contributions

Conceived and designed the experiments: QC, and QW. Performed the experiments: MK, BL, and YJ. Analyzed the data: QC and MK. Wrote the paper: MK and QC.

## Conflict of Interest Statement

The authors declare that the research was conducted in the absence of any commercial or financial relationships that could be construed as a potential conflict of interest. The reviewer GN-V and handling Editor declared their shared affiliation.

## Footnotes

1 http://primerexplorer.jp/elamp4.0.0/index.html

## Supplementary Material

FIGURE S1 Detection of *P. infestans* using the optimized LAMP system. **(A)** LAMP assay and visual inspection using a fluorescent metal indicator (calcein) observed by the naked eye. Positive reactions turned green in the presence of calcein. **(B)** The LAMP assay was evaluated using 2% agarose gel electrophoresis. The ladder-like bands indicate a positive reaction for *P. infestans.* Lane M, DL2000 DNA markers, tube (+) represents a positive reaction; tube (-) represents the negative control. The same results were obtained in three repeat assessments.

FIGURE S2 Specificity detection of PCR primers based on *Ypt1* gene designed for *P. infestans.* Lane M, 2000 bp DNA Marker; Lane 1, negative control; Lane 2–5: *P. infestans;* Lane 6, *P. cactorum;* Lane 7, *P. nicotianae;* Lane 8, *P. capsici;* Lane 9, *Botrytis cinerea;* Lane 10, *Fusarium oxysporum;* Lane 11, *Colletotrichum gloeosporioides.* The same results were obtained in three repeat assessments.

FIGURE S3 Sensitivity of real-time PCR product by gel electrophoresis. Lane M, 500 bp DNA ladder Marker; Lane 1, 1.28 × 10^2^ ng **μ**L^-1^; Lane 2, 1.28 × 10 ng **μ**L^-1^; Lane 3, 1.28 ng μL^-1^; Lane 4, 1.28 × 10^-1^ ng **μ**L^-1^; Lane 5, 1.28 × 10^-2^ ng **μ**L^-1^; Lane 6, 1.28×10^-3^ng **μ**L^-1^; Lane 7, 1.28 × 10^-4^ ng **μ**L^-1^; Lane 8, Negative control. The same results were obtained in three repeat assessments.

FIGURE S4 LAMP detection of artificial infection of leaves. The potato leaves were artificially infected with *P. infestans*, and were incubated at room temperature for different times. **(A)** The DNA was extracted with rapid DNA method and was then subjected to LAMP assay and **(B)** the products were analyzed on 2% agarose gel electrophoresis. Lane 1, 1 h; Lane 2, 2 h; Lane 3, 4 h; Lane 4, 8 h; Lane 5, 16 h; Lane 6, 32 h; Lane 7, positive control; Lane 8, Healthy plant; Lane M, 2000-bp DNA marker. The same results were obtained in three repeat assessments.
