# Supplementary figures and images for: Evaluation of Different PCR-Based Assays and LAMP Method for Rapid Detection of Phytophthora infestans by Targeting the Ypt1 Gene
Source: Front Microbiol. 2017 Oct 5;8:1920. doi: 10.3389/fmicb.2017.01920 (PMC5633602; doi:10.3389/fmicb.2017.01920)

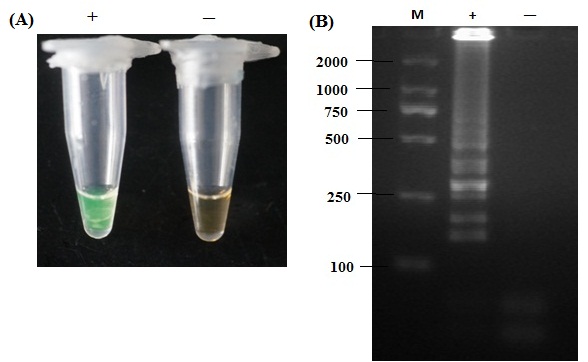

Supplement: FIGURE S1 — Detection of P. infestans using the optimized LAMP system. (A) LAMP assay and visual inspection using a fluorescent metal indicator (calcein) observed by the naked eye. Positive reactions turned green in the presence of calcein. (B) The LAMP assay was evaluated using 2% agarose gel electrophoresis. The ladder-like bands indicate a positive reaction for P. infestans. Lane M, DL2000 DNA markers, tube (+) represents a positive reaction; tube (-) represents the negative control. The same results were obtained in three repeat assessments. [file Image_1.JPEG]

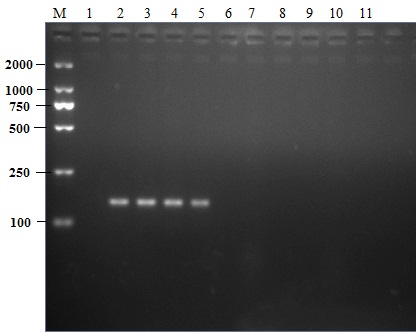

Supplement: FIGURE S2 — Specificity detection of PCR primers based on Ypt1 gene designed for P. infestans. Lane M, 2000 bp DNA Marker; Lane 1, negative control; Lane 2–5: P. infestans; Lane 6, P. cactorum; Lane 7, P. nicotianae; Lane 8, P. capsici; Lane 9, Botrytis cinerea; Lane 10, Fusarium oxysporum; Lane 11, Colletotrichum gloeosporioides. The same results were obtained in three repeat assessments. [file Image_2.JPEG]

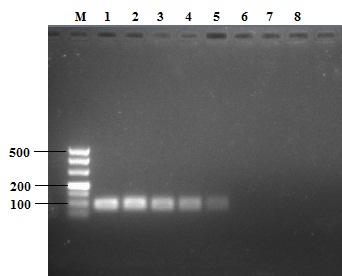

Supplement: FIGURE S3 — Sensitivity of real-time PCR product by gel electrophoresis. Lane M, 500 bp DNA ladder Marker; Lane 1, 1.28 × 10^2^ ng μL-1; Lane 2, 1.28 × 10 ng μL-1; Lane 3, 1.28 ng μL-1; Lane 4, 1.28 × 10^-1^ ng μL-1; Lane 5, 1.28 × 10^-2^ ng μL-1; Lane 6, 1.28×10^-3^ng μL-1; Lane 7, 1.28 × 10^-4^ ng μL-1; Lane 8, Negative control. The same results were obtained in three repeat assessments. [file Image_3.JPEG]

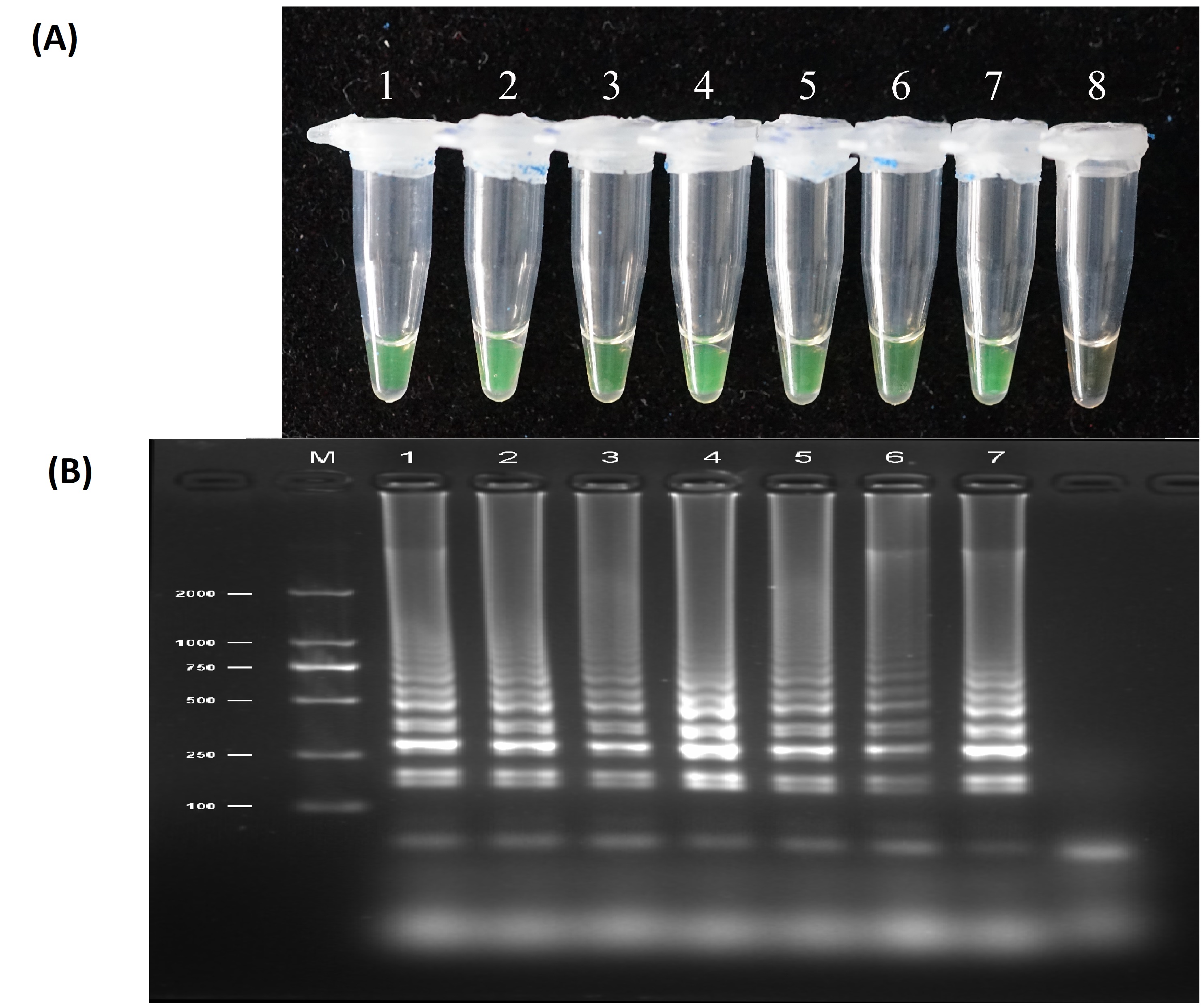

Supplement: FIGURE S4 — LAMP detection of artificial infection of leaves. The potato leaves were artificially infected with P. infestans, and were incubated at room temperature for different times. (A) The DNA was extracted with rapid DNA method and was then subjected to LAMP assay and (B) the products were analyzed on 2% agarose gel electrophoresis. Lane 1, 1 h; Lane 2, 2 h; Lane 3, 4 h; Lane 4, 8 h; Lane 5, 16 h; Lane 6, 32 h; Lane 7, positive control; Lane 8, Healthy plant; Lane M, 2000-bp DNA marker. The same results were obtained in three repeat assessments. [file Image_4.JPEG]
